# Bridging the Under-Five Mortality Gap for Africa in the Era of Sustainable Development Goals: An Ordinary Least Squares (OLS) Analysis

**DOI:** 10.29024/aogh.9

**Published:** 2018-04-30

**Authors:** Michael Acheampong, Chukwudi Ejiofor, Abraham Salinas-Miranda, Foday M. Jaward, Michael Eduful, Qiuyan Yu

**Affiliations:** 1 School of Geosciences, University of South Florida, 4202 E. Fowler Avenue, NES 107 Tampa, FL 33620-5550, US; 2 College of Public Health, University of South Florida, 13201 Bruce B. Downs Blvd. MDC 56, Tampa, FL 33612, US

## Abstract

**Background:**

While Africa achieved significant progress in reducing under-five mortality rate (U5MR) in the MDGs era, it did not achieve the set target and still has the highest average of 81 deaths per 1000 live births compared to a global average of 43 deaths. The SDG number 3 has set a new target of reducing U5MR to 25 deaths per 1000 births in the world, which serves a huge challenge, especially for Africa. Socioeconomic inequities that remain unaddressed across countries account for Africa’s high U5MR. Unless there is adequate prioritization of important socioeconomic, healthcare, and environmental factors, the new SDGs target will be hindered.

**Objectives:**

In this study, our primary objective was to analyse and assess factors that account most for the U5MR inequities between Africa and the rest of the world.

**Methods:**

We conducted a series of ordinary least squares (OLS) regression-based prioritization analysis of socioeconomic, healthcare, and environmental variables from 43 African countries in a pool of 109 countries from around the world to understand the most important factors that account most for the high U5MR in Africa.

**Findings:**

The results suggest that the most critical category for bridging the U5MR gap with the rest of the world is improved healthcare access. However, with all categories examined together, the OLS regression showed that the most important factors that accounted for Africa’s high U5MR compared with the rest of the world were, in order: fertility rate, access to improved water, total health expenditure per capita, access to improved sanitation, and female employment rate.

**Conclusions:**

The findings reveal that Africa will significantly benefit from interventions geared towards both the treatment and prevention of acute infectious diseases in the form of providing affordable maternal healthcare services, as well as providing access to improved drinking water and sanitation.

## Introduction

Globally, the rates of under-five mortality rates (U5MR) fell by 53% between 1990 (91 per 1,000) and 2015 (43 per 1,000) [1], with at least 50% decline in U5MR in all WHO regions. Although this has been a great achievement, the target for the fourth Millennium Developmental Goal (MDG) of a two-thirds reduction in U5MR was not achieved. The sub-Saharan Africa region demonstrated tremendous progress from the baseline, but the end result was still insufficient due to a significant proportion of countries that remained with high to very high child mortality [2]. A faster reduction was very challenging for sub-Saharan countries with poor prioritization of social determinants and deficient allocation of resources.

As of 2015, which marked the end of MDGs, 5.9 million children were estimated to have died before the age of five, with Africa bearing the greatest burden [1]. Sub-Saharan Africa, which has had an impressive yearly decline in its rates of U5MR over the past two decades, still has the highest rates of childhood mortality worldwide (1 in 9 children) [1]. Despite having just 11% of the world’s population, sub-Saharan Africa accounts for half of the global burden of maternal, newborn, and child deaths. On the bright side, some African countries (Liberia, Rwanda, Malawi, and Madagascar, to mention but a few) were able to achieve the MDG 4 target, reducing their U5MR by 67.5%, 65.4%, 63.6%, and 61.8%, respectively [1,3]. The decline in U5MR in Africa was achieved by addressing the direct and indirect factors that led to the unfavorable outcomes, thereby reducing the rates of major causes of childhood mortality. They include malnutrition, HIV/AIDS, and acute illnesses such as diarrheal diseases, pneumonia, malaria, and vaccine-preventable diseases [2,3].

Various factors have been shown to affect the health outcomes of children in Africa. Socioeconomic conditions play an important role in both availability of adequate health care and access to these resources. Poor antenatal and postnatal care, limited knowledge on the part of mothers due to low level of education and lack of empowerment, and inadequate or inconsistent investment into services addressing child health by these countries all contribute to the slow progress in the decline of U5MR [3,4]. African countries that have made the most progress have made efforts towards instituting targeted evidence-based interventions addressing access to health care and strengthened their health care systems at all levels [3,5]. Using available evidence-based interventions and adapting them to the different communities based on their individual priorities would have the greatest impact as we move towards achieving the new sustainable development goals (SDGs).

A previous study by Acheampong et al. [6]. has already outlined global priorities towards achieving the SDG goal of reducing U5MR to 25 deaths per 1,000 children. However, it is important to understand that in-country and regional peculiarities may derail one-size-fits-all prioritisation approach. To address this for Africa, it is important to identify which factors best explain the wide U5MR gap between Africa and the rest of the world. Also, identifying the degree to which each factor influences the primary outcome will help these countries in prioritizing their interventions based on need. The results of this study may help as a guide for policymakers, funding agencies, and program planners.

## Methods

### Design and Data Sources

This is a population-based, cross-sectional study using extant data from the following sources: the World Bank’s World Development Indicators (WDI) database [7]; the World Health Organization (WHO) Global Health Expenditure Database [8]; the Central Intelligence Agency (CIA) World Factbook [9]; and the United Nations Educational, Scientific, and Cultural Organization [10]. Appendix 1 shows the list of variables and their corresponding sources. Our sample consisted of 109 countries for which complete data were available, as in Acheampong et al. [6]. These countries were from all the six WHO world regions, with 43 of them from Africa. Table 1 presents the list of African countries included.

**Table 1:** List of African countries and their 2010 under-five mortality rates used in the study.

Country	Under-5 mortality rate	Country	Under-5 mortality rate	Country	Under-5 mortality rate

Algeria	27.4	Gabon	63	Mozambique	103.8
Angola	182.5	Gambia	81.4	Namibia	55.4
Benin	111.6	Ghana	74.7	Niger	123.6
Botswana	60.3	Guinea	111.9	Nigeria	130.3
Burkina Faso	113.5	Guinea-Bissau	115.9	Rwanda	64.2
Burundi	98.8	Kenya	63.6	Senegal	64.8
Cameroon	106	Lesotho	101.5	Sierra Leone	160.2
Cape Verde	27.9	Liberia	89.3	South Africa	54.4
Central African Republic	150.9	Madagascar	60.3	Sudan	80.2
Chad	160.1	Malawi	92	Tanzania	62.3
Comoros	86	Mali	136.6	Togo	90.9
Congo, Dem. Rep.	116.1	Mauritania	97.8	Tunisia	17.4
Cote d’Ivoire	110.1	Mauritius	15.2	Uganda	79.5
Egypt	29	Morocco	33.1	Zambia	84.8
Equatorial Guinea	110.9				
Ethiopia	75.7				

**Figure 1:**
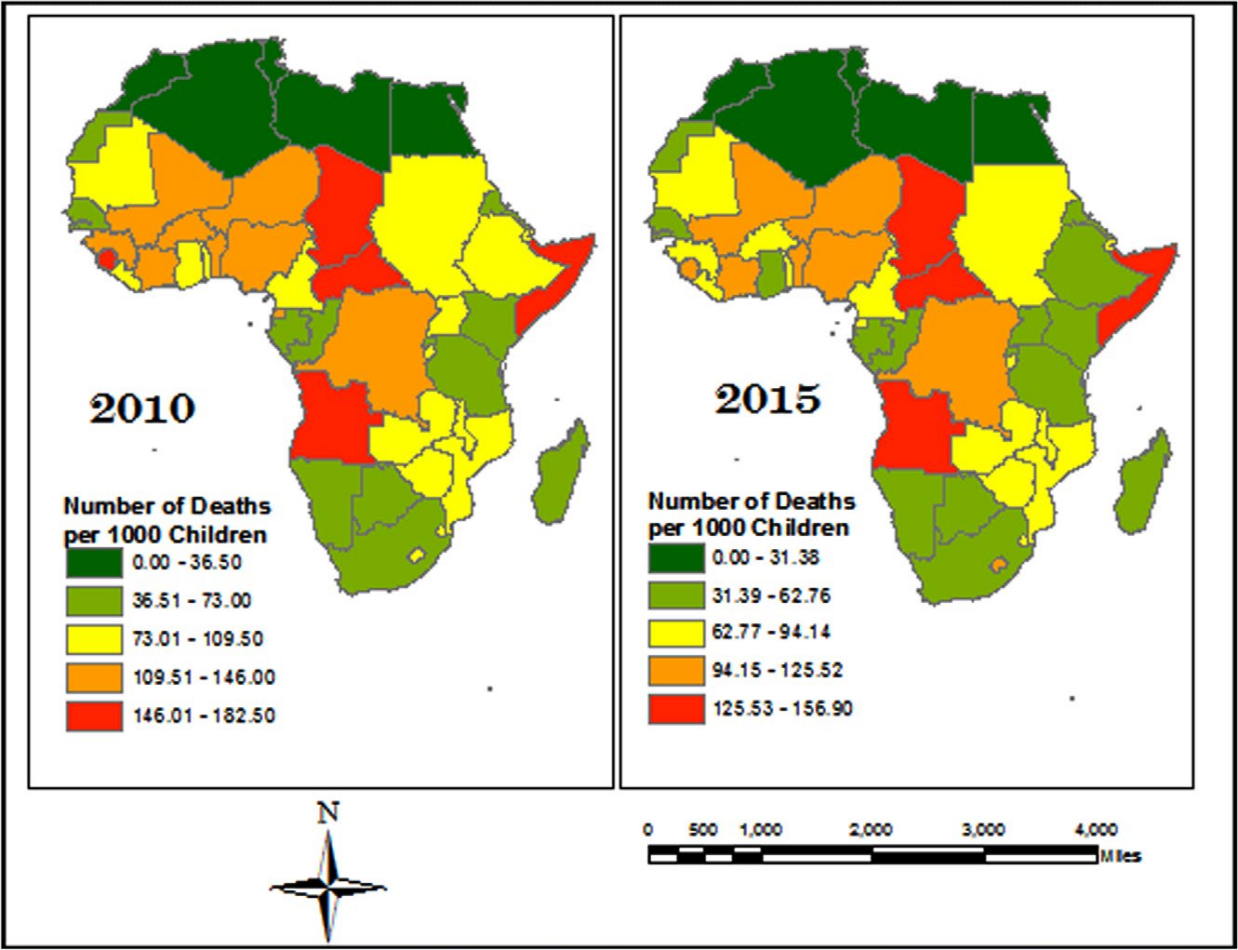
Equal Interval Distribution of U5MR across Africa in 2010 and 2015.

Due to lack of recent data for most variables under consideration, data from 2010 was used because it had a comprehensive record of data. Figure 1, developed by the authors using ArcGIS 10.2, shows that the U5MR distribution within Africa has seen little change between 2010 and 2015. Therefore, using 2010 data would be sufficient to depict relationships that currently prevails.

### Variables

Following in the steps of Acheampong et al. [6], we conducted our analysis using 13 variables that were grouped under social, economic, healthcare accessibility, and environmental categories (Table 2). We also used a fourteenth variable, Africa dummy, which assigned a value of 1 to all African countries and 0 to all others. The relevance of the dummy variable is to assess the extent to which the various factors account for the exceptionally high average U5MR that is observed in Africa vis-à-vis the rest of the world. Appendix 1 presents details on guiding principles for selecting each variable.

**Table 2:** Categories of Independent Variables.

Social	Economic	Healthcare Access	Sanitary/Environmental

Total Fertility Rate	GNI per Capita	Per Capita Total Expenditure on Health	Per Cent Population with Access to Improved Sanitation
Adolescent Fertility Rate	Total Female Employment to Population Ratio	Out-of-pocket Expenditure as a Per Cent of Total Health Expenditure	Per Cent Population with Access to Improved Drinking Water Source
Total Adult Literacy Rate	Per Cent Population Living under National Poverty Line	Government Expenditure on Health as a Per Cent of Total Health Expenditure	
Female Adult Literacy Rate			
Rural Population			

### Specifications of Models

We used a series of ordinary least squares (OLS) models to determine factors that can explain the unusually high U5MR in Africa in relation to the rest of the world. We carried out data transformation where necessary to fulfill the assumptions of OLS such as linearity, normality, homogeneity of variances, and homoscedasticity [11,12,13].

### Multiple Regression Analyses

We carried out four different multiple regression models for each category of variables, with a dummy variable for Africa, in order to determine how each category explained Africa’s high U5MR vis-à-vis global rates. However, before considering all the variables in each category in a regression model, we examined their individual abilities to explain the significant difference between Africa and other parts of the world in terms of U5MR. With these models, we hoped to be able to illuminate whether it is social, economic, healthcare accessibility, or environmental factors that accounted for the high disparities between Africa and the rest of the world. We constructed another that combined all the variables in a single, full model to examine their combined effects on Africa’s U5MR. In a subsequent model, we addressed the issues of possible multicollinearity and dropped superfluous variables to see if there was a change in effect. Finally, we constructed our last model that ranked variables with the greatest effect when combined together. Below, we have summarized the formulas for the models, starting with individual categories and their respective variables. It should, however, be noted that all models are a subset of the full model.

Social Factors (Category 1)

*U = α* + *βnC*1*Vn* + *βA* + *ɛ*

*U = α* + *β*1*C*1*V*1 + *β*2*C*1*V*2 + *β*3*C*1*V*3 + *β*4*C*1*V*4 + *β*5*C*1*V*5 + *βA* + *ɛ*

Economic Factors (Category 2)

*U = α* + *β*n*C*2*Vn* + *βA* + *ɛ*

*U = α* + *β*6*C*2*V*1 + *β*7*C*2*V*2 + *β*8*C*2*V*3 + *βA* + *ɛ*

Healthcare Access Factors (Category 3)

*U = α* + *β*n*C*3*Vn* + *βA* + *ɛ*

*U = α* + *β*9*C*3*V*1 + *β*10*C*3*V*2 + *β*11*C*3*V*3 + *βA* + *ɛ*

Environmental Factors (Category 4)

*U = α* + *β*n*C*4*Vn* + *βA* + *ɛ*

*U = α* + *β*12*C*4*V*1 + *β*13*C*4*V*2 + *βA* + *ɛ*

Full Model

*U = α* + *β*1*C*1*V*1 + … + *β*6*C*2*V*1 + … + *β*9*C*3*V*1 + … + *β*13*C*4*V*2 + *βA* + ɛ

Where:

*U* = the U5MR of any given country

*α* = the Y-intercept

*A* = Africa Dummy

*β* = slope of the Africa Dummy

*β*n = Slope of a predictor variable

*β*1 … *β*13 = slope of each of the 13 predictor variables studied

*CxVn* = Nth variable in *x*th category

*C*1*V*1 = Total Fertility Rate

*C*1*V*2 = Adolescent Fertility Rate

*C*1*V*3 = Female Adult Literacy Rate

*C*1*V*4 = Total Adult Literacy Rate

*C*1*V*5 = Rural Population

*C*2*V*1 = GNI per Capita

*C*2*V*2 = Total Female Employment to Population Ratio

*C*2*V*3 = Per Cent Population Living under National Poverty Line

*C*3*V*1 = Per Capita Total Expenditure on Health

*C*3*V*2 = Out-of-pocket Expenditure as a Per Cent of Total Health Expenditure

*C*3*V*3 = Government Expenditure on Health as a Per Cent of Total Health Expenditure

*C*4*V*1 = Per Cent Population with Access to Improved Sanitation

*C*4*V*2 = Per Cent Population with Access to Improved Drinking Water Source

*ɛ* = the error term

## Results

### Summary of Descriptive Statistics for All Independent Variables (IVs)

Table 3 below presents a summary of descriptive statistics for the IVs examined. The coefficient of variation indicates that total expenditure on health exhibits the highest variation among African countries.

**Table 3:** Descriptive statistics for independent variables in the study.

Variable**	Minimum	Maximum	Mean	Standard Deviation	Coefficient of Variation (Mean/SD)

Total Fertility	1.52	7.58	4.87	1.27	0.26
Adolescent Fertility	10.73	210.37	105.12	42.81	0.41
Adult Literacy	25.31	94.23	62.41	19.14	0.31
Female Literacy	12.19	92.18	54.83	22.88	0.42
Rural Per Cent	14.30	89.36	61.98	15.89	0.26
Income per Capita	560.00	26790.00	4042.33	5284.64	1.31
Female Employment	11.80	86.40	57.14	18.31	0.32
Poverty Level	8.00	76.80	46.21	15.90	0.34
Gov. Expenditure	1.84	20.08	10.14	3.94	0.39
Total Expenditure	11.90	896.19	122.25	192.95	1.58
Personal Expenditure	7.45	88.15	39.03	21.07	0.54
Sanitation	9.50	92.70	34.12	21.48	0.63
Water	44.00	99.00	69.14	15.89	0.23

Note: Number of observations for all variables = 43. **Abbreviations below are used in all tables throughout the paper. Total Fertility = Total Fertility Rate; Adolescent Fertility = Adolescent Fertility Rate; Adult Literacy = Total Adult Literacy Rate; Female Literacy = Female Adult Literacy Rate; Rural Per Cent = Per Cent Rural Population; Income per Capita = Gross National Income per Capita; Female Employment = Total Female Employment to Population Ratio; Poverty Level = Per Cent Population Living under National Poverty Level; Government Expenditure = Per Cent Government Expenditure on Health Per Capita; Total Expenditure = Total Expenditure on Health; Personal Expenditure = Per Cent Out-of-Pocket Expenditure; Sanitation = Per Cent Population with Access to Improved Sanitation Facilities; Water = Per Cent Population with Access to Improved Drinking.

Table 4 below shows the results of the explanatory strength of social factors on the U5MR difference between Africa and the rest of the globe. As seen, there is a high correlation between all the individual social factors considered and the DV, and it reveals that they all have a significant influence on Africa’s high U5MR, with a statistically significant Africa dummy variable (Models 1-A through 1-E, all showing either *p* < 0.05 or *p* < 0.001). In model 1-F, we constructed a multivariate model that considered all the social variables together. The adjusted R^2^ for the model was 0.86. This is an indication that about 86% of the variability in U5MR differences across countries can be explained by differences in social factors. Total Fertility Rate, Female Adult Fertility Rate, and Per Cent Rural Population demonstrated high positive associations (*p* < 0.001), On the other hand, Total Adult Literacy Rate and Female Adult Literacy Rate were statistically insignificant in the model. However, in the full social category model, Africa dummy variable becomes statistically insignificant, meaning that the unusually high U5MR in Africa cannot necessarily be explained by the difference in their status as opposed to the rest of the world as far as these factors are concerned.

**Table 4:** Effect of Social Factors on Under-5 Mortality in Africa.

	Model 1-A		Model 1-B		Model 1-C		Model 1-D		Model 1-E		Model 1-F	

Total Fertility	1.79	***									0.73	***
	(0.16)										(0.20)	
Adolescent Fertility			0.14	***							0.06	***
			(0.01)								(0.02)	
Female Literacy					–0.43	***					0.11	
					(0.06)						(0.29)	
Adult Literacy							–0.51	***			–0.22	
							(0.06)				(0.33)	
Rural Per Cent									0.57	***	0.29	***
									(0.06)		(0.05)	
Africa Dummy	0.37	*	0.38	*	0.84	***	0.75	***	1.23	***	0.12	
	(0.17)		(0.18)		(0.18)		(0.18)		(0.14)		(0.16)	
Constant	1.29	***	1.47	***	3.65	***	3.93	***	2.87	***	2.03	***
	(0.14)		(0.13)		(0.16)		(0.19)		(0.09)		(0.30)	
F-Statistic	185.9	***	174.1	***	121.6	***	122.8	***	134.9	***	109.6	***
Adj. R^2^	0.77		0.76		0.69		0.69		0.71		0.86	

Notes: Standard errors are reported in parenthesis. Entries in the table are standardized regression coefficients. Number of observations = 109. *p < 0.05; **p < 0.01; ***p < 0.001.

### Model Results for Economic Factors

Table 5a below shows the results of the explanatory strength of economic factors on the U5MR difference between Africa and the rest of the world. There is a high correlation between all the individual economic factors considered and the DV, and they exert a significant influence on Africa’s high U5MR, with a statistically significant Africa dummy variable (Models 2-A through 2-C; *p* < 0.001). In model 2-D, we constructed a multivariate model that considered all the economic variables together. With a high statistically significant Africa dummy variable (*p* < 0.001) in the full model, the adjusted R^2^ of 0.77 indicates that up to 77% of differences in U5MR between Africa and the rest of the world can be accounted for by economic factors. Within the model 2-D, GNI per capita displayed a strong negative association with the DV (*p* < 0.001), which indicates that the U5MR for countries decrease while their GNI per capita increase. Total Female Employment to Population Ratio also showed a significant positive relationship with the DV, albeit weakly (*p* < 0.1), whereas Per Cent Population Living under National Poverty Line showed no statistical significance.

**Table 5a:** Effect of Economic Factors on Under-5 Mortality in Africa.

	Model 2-A		Model 2-B		Model 2-C		Model 2-D	

Income per Capita	–0.64	***					–0.66	***
	(0.06)						(0.07)	
Female Employment			0.00				–0.01.	
			(0.01)				(0.00)	
Poverty Level					0.43	***	0.01	
					(0.11)		(0.09)	
Africa Dummy	0.73	***	1.78	***	1.19	***	0.77	***
	(0.15)		(0.18)		(0.22)		(0.17)	
Constant	8.66	***	2.58	***	3.27	***	9.07	***
	(0.55)		(0.23)		(0.20)		(0.62)	
F-Statistic	182.3	***	56.7	***	71.9	***	93.1	***
Adj. R^2^	0.77		0.51		0.57		0.77	

Notes: Standard errors are reported in parenthesis. Entries in the table are standardized regression coefficients. Number of observations = 109. .*p* < 0.1; *p < 0.05; **p < 0.01, ***p < 0.001.

### Model Results for Healthcare Accessibility Factors

Table 5b below shows the results of the explanatory strength of factors affecting access to healthcare on the U5MR difference between Africa and the rest of the globe. There is a high correlation between all these factors considered individually and the DV, and they exert a significant influence on Africa’s high U5MR, with a statistically significant Africa dummy variable (Models 3-A through 3-C; *p* < 0.001). Model 3-D, however, shows a multivariate model that contains all the healthcare accessibility variables together. The adjusted R^2^ of 0.84 indicates that up to 84% of differences in U5MR between countries can be accounted for by healthcare accessibility factors. The high statistically significant Africa dummy variable (p < 0.001) in the full model indicates that healthcare accessibility factors are critical in explaining the huge difference of U5MR between Africa and the rest of the world. Within the model 3-D, Per Capita Total Expenditure on Health displayed the strongest negative association with the DV (*p* < 0.001). Government Expenditure on Health as a Per Cent of Total Health Expenditure also showed a significant negative relationship with the DV (*p* < 0.05), whereas Out-of-pocket Expenditure as a Per Cent of Total Health Expenditure showed no statistical significance.

**Table 5b:** Effect of Healthcare Accessibility Factors on Under-5 Mortality in Africa.

	Model 3-A		Model 3-B		Model 3-C		Model 3-D	

Gov. Expenditure	–0.12	***					–0.04	*
	(0.02)						(0.02)	
Total Expenditure			–0.47	***			–0.42	***
			(0.03)				(0.04)	
Personal Expenditure					0.13	***	–0.01	
					(0.02)		(0.02)	
Africa Dummy	1.66	***	0.86	***	1.69	***	0.93	***
	(0.14)		(0.12)		(0.15)		(0.12)	
Constant	3.88	***	5.43	***	1.39	***	5.63	***
	(0.19)		(0.21)		(0.22)		(0.42)	
F-Statistic	113.8	***	267.0	***	94.3	***	139.8	***
Adj. R^2^	0.68		0.83		0.63		0.84	

Notes: Standard errors are reported in parenthesis. Entries in the table are standardized regression coefficients. Number of observations = 109. *p < 0.05; **p < 0.01; ***p < 0.001.

### Effects of Environmental Factors

Table 6 below shows the results of the explanatory strength of environmental factors on the U5MR difference between Africa and the rest of the world. There is a high negative correlation between both environmental variables considered individually and the DV. This establishes that they exert a significant influence on Africa’s high U5MR, with a statistically significant Africa dummy variable (Models 4-A and 4-B; *p* < 0.001). Model 4-C shows a multivariate model that contains both variables considered together in a single model. The adjusted R^2^ of 0.84 indicates that up to 84% of differences in U5MR between countries can be accounted for by environmental factors. Meanwhile, the statistically significant Africa dummy variable (*p* < 0.1) in the full model, while weak, shows that they are important in explaining Africa’s unusually high U5MR, because there is a significant difference between Africa and the rest of the world as far as these factors are concerned. Within the model 4-C, both Per Cent Population with Access to Improved Sanitation and Per Cent Population with Access to Improved Drinking Water Source Per Capita Total Expenditure on Health displayed the strongest negative association with the DV (*p* < 0.001).

**Table 6:** Effect of Environmental Factors on Under-5 Mortality in Africa.

	Model 4-A		Model 4-B		Model 4-C	

Water	–0.66	***			–0.42	***
	(0.05)				(0.06)	
Sanitation			–0.53	***	–0.28	***
			(0.04)		(0.05)	
Africa Dummy	0.65	***	0.30.		0.27.	
	(0.13)		(0.16)		(0.14)	
Constant	4.36	***	3.73	***	4.32	***
	(0.15)		(0.12)		(0.13)	
F-Statistic	236.7	***	206.8	***	208.0	***
Adj. R^2^	0.82		0.79		0.85	

Notes: Standard errors are reported in parenthesis. Entries in the table are standardized regression coefficients. Number of observations = 109. .*p* < 0.1; ***p < 0.001.

### Comparing Explanatory Powers of the Different Categories

Table 7 below presents a rank ordering of the *t*-statistic of the Africa dummy variable in the different models constructed for the different categories of variables. This is done to show which of the factors may be more important to consider in helping Africa bridge the U5MR gap with the rest of the world, all things being equal. From the table, it shows that differential access to healthcare (7.71) has the strongest effect on the wide difference in U5MR between Africa and other world regions. Economic factors (4.56) come second on the list, and environmental factors (1.98) follow in third. Ranked fourth is social category (0.76), which showed no statistically significant relevance in explaining Africa’s high U5MR compared to the rest of the world.

**Table 7:** Rank Ordering Categories.

Ind. Variable	*β*	SE	*t*-statistic	

Africa*Healthcare Access	0.93	0.12	7.71	***
Africa*Economic	0.77	0.17	4.56	***
Africa*Environmental	0.28	0.14	1.98.	
Africa*Social	0.12	0.16	0.76	

Number of observations = 109. .*p* < 0.10; *p < 0.05; **p < 0.01; ***p < 0.001.

In Table 8 below, we present a model with all variables from the different categories considered, as well as the Africa dummy variable in Model 5-A. We also present the most parsimonious model (Model 5-B) for comparison. Both models produced similar outcomes, with an adjusted R^2^ value of 0.91. The results suggest that models 5-A and 5-B explained approximately 91% of the inter-country differences in U5MR. However, there are two major differences between the two models. First, the first model is less robust than the second in explaining inter-country U5MR differentials, with F-statistics of 74.2 (*p* < 0.001) and 117.0 (*p* < 0.001), respectively. Secondly, the Africa dummy variable is statistically insignificant in the model 5-A, whereas it becomes significant in 5-B (*p* < 0.1). This means that the first model cannot explain the magnitude of differences between Africa and the rest of the world, while the second can even though weakly (*p* < 0.1). Model 5-B, therefore, answers the question as to which factors explain why Africa has a significantly higher U5MR as compared to other regions around the globe. Seven variables, in addition to the Africa dummy, were statistically significant in model 5-B. These variables were: Per Capita Total Expenditure on Health with a negative relationship (*p* < 0.001); Total Fertility Rate and Adolescent Fertility Rate with positive relationships (*p* < 0.01); Per Cent Population with Access to Improved Sanitation and Per Cent Population with Access to Improved Drinking Water Source showed a negative relationships (*p* < 0.05); GNI per Capita with a positive relationship (*p* < 0.1); and Total Female Employment to Population Ratio with a negative relationship (*p* < 0.1).

**Table 8:** Effects of all factors on U5MR in Africa.

Ind. Variable	Model 5-A		Model 5-B		Model 5-C	

Total Fertility	0.55	**	0.47	**		
	(0.20)		(0.18)			
Adolescent Fertility	0.04	**	0.04	**	0.05	***
	(0.01)		(0.01)		(0.01)	
Female Literacy	0.30					
	(0.25)					
Adult Literacy	–0.28					
	(0.30)					
Rural Percent	0.08		0.09			
	(0.06)		(0.06)			
Income per Capita	0.14		0.20.		0.12	
	(0.13)		(0.10)		(0.10)	
Female Employment	–0.01.		–0.00.		–0.00.	
	(0.00)		(0.00)		(0.00)	
Poverty Level	0.02					
	(0.07)					
Gov. Expenditure	–0.01					
	(0.02)					
Total Expenditure	–0.23	*	–0.28	***	–0.27	***
	(0.09)		(0.07)		(0.07)	
Personal Expenditure	0.01					
	(0.02)					
Water	–0.14	*	–0.15	*	–0.24	***
	(0.07)		(0.07)		(0.06)	
Sanitation	–0.13	*	–0.12	*	–0.11	*
	(0.06)		(0.05)		(0.05)	
AFRICA Dummy	0.23		0.23		0.30	*
	(0.15)		0.13		(0.13)	
Constant	2.85	*	2.61	**	3.80	***
	(1.11)		(0.79)		(0.69)	
F-statistic	74.2	***	117.0	***	140.2	***
Adj. R^2^	0.91		0.91		0.90	

Notes: Standard errors are reported in parenthesis. Entries in the table are standardized regression coefficients. Number of observations = 109. .*p* < 0.1; *p < 0.05; **p < 0.01; ***p < 0.001.

To examine which of the variables was of the most importance, we constructed a final model (Model 5-C). In Model 5-C, we dropped what we found to be redundant variables in model 5-B through a variance inflation factor (VIF) analysis, along with variables found to be statistically insignificant. In total, we dropped Total Fertility Rate and Per Cent Rural Population in model 5-C. Model 5-C offered an even stronger model with F-statistic of 140.2 (*p* < 0.001), with an adjusted R^2^ of 0.90. In Table 9, we rank ordered the variables from Model 5-C based on the absolute values of their *t*-statistics, in order of importance.

**Table 9:** Rank Ordering Variables according to their effects in Model 5-C.

Ind. Variable	*β*	SE	*t*-statistic	

Adolescent Fertility	0.05	0.01	4.12	***
Water	–0.24	0.06	–4.10	***
Total Expenditure	–0.27	0.07	–3.76	***
Sanitation	–0.11	0.05	–2.04	*
Female Employment	–0.00	0.00	–0.07.	

Notes: Constant = 3.80, Adj. R^2^ = 0.90, F = 144.2, *p* < 0.001. .*p* < 0.1; *p < 0.05; ***p < 0.001.

In the ranking, Adolescent Fertility Rate had the strongest effect on the difference in U5MR between African countries and the rest of the world. Per Cent Population with Access to Improved Drinking Water Source was second, and Per Capita Total Expenditure on Health with a negative relationship followed. Following that was Per Cent Population with Access to Improved Sanitation, and then, Total Female Employment to Population Ratio in that order. GNI per Capita is absent because it became statistically insignificant in model 5-C.

## Discussion

The analyses above suggest that all the categories of factors, apart from the social factors, play a significant role in the disparity between Africa and the rest of the world as far as U5MR is concerned. In theory, Table 7 suggests that Africa may fare better in bridging the gap with the rest of the world in terms of U5MR with increased investment in improved healthcare access to populations, with everything held constant. Increasing expenditure on healthcare, especially from government, may directly translate into improved healthcare access for mothers in developing countries who may have less money to afford healthcare (Model 3-D). Our analysis also suggests that Africa may achieve no significant success in bridging the U5MR gap with the other world regions if it expends its limited resource just on improving its standing in the social factors included in the analysis, with all things being equal.

Because it is likely unrealistic to pursue only one set of factors in a society without impacting others, our analysis in the subsequent models revealed that with all factors combined, the difference in Adolescent Fertility Rate between Africa and the rest of the world may be most accounting for the huge disparities in terms of U5MR. Currently, Africa has the highest adolescent birth rate of about 120 per 1,000 adolescent women, far above Latin America and the Caribbean, which is the second highest with about 80. The continent also has the ten countries with the highest adolescent birth rates in the world [14]. Identifying this as the most critical factor, with all things considered, is in tandem with the order of global priorities, as Acheampong et al. [6]. already found. This is not a surprise because early childbearing reduces the education and employment opportunities, and stable marriages, that adolescent may have had otherwise, all compounding their vulnerability and that of their children to easily preventable fatalities [6,14,15,16].

Our analyses show that while global priorities remain relevant for Africa, there are instances where nuances have to be understood as far as the order of priorities is concerned. The global analysis in Acheampong et al. [6]. found that increased expenditure on healthcare was more relevant than access to improved water, in order to achieve the SDG targets around the globe. However, in this study, we found that as far as Africa is concerned, improving water quality usurps increased expenditure in importance in the pursuit of bridging the U5MR gap. After Total Expenditure on Healthcare, we find that access to improved sanitation is next in order of priorities to improve the performance of Africa in reducing the deaths of children under five years old.

These findings are not surprising given that nearly 70% of all child deaths worldwide are attributed to acute infectious diseases that can be treated by vaccination [5,17]. According to statistics from the UNICEF [18], diarrhea alone accounts for about 11% of all under-five deaths worldwide, with about 90% of these total deaths occurring sub-Saharan Africa. For instance, in 2011, cholera outbreaks in West and Central Africa claimed nearly 2,500 lives, the majority of whom were below five years old [19]. The CDC [20] concluded that many African countries face a dual challenge of treatment and prevention with regards to cholera outbreaks. The treatment challenge exists in the form of lack of access to the basic healthcare that can contain cholera fatalities, while the prevention challenge exists in the lack of access to improved water and sanitation systems [20]. This statement confirms our three most significant factors identified after the number of adolescent child births.

As revealed by many other studies [21,22], we found female employment to have a negative association with U5MR. This is because income earning opportunities reduce the barrier of access to healthcare for themselves and their children [21]. In addition, additional income implies that mothers are capable of providing better living conditions for their children [23].

### Policy and Program Implications

This study reaffirmed that global priorities as identified by Acheampong et al. [6]. are highly relevant for Africa. For Africa to bridge the U5MR gap with the rest of the world, our study suggests that policymakers and international funding agencies should channel resources to increase educational programs that target mothers, especially for adolescents who suffer the highest rate of under-five deaths. In addition, policies and resources should focus on dealing with the dual problem of disease treatment and prevention through improving access to basic health services and improving access to improved drinking water and sanitation, respectively. While it may not be easily subjected to intervention in the short term, it is important to also bear in mind that improving gender equality in employment access will also help bridge the U5MR gap between Africa and the rest of the globe.

## Study Limitations

It is important to acknowledge some of the limitations that are inherent in our analysis. Most prominently, even though we strived to include as many countries as possible in the research for a more comprehensive outcome, it is imperative that we emphasize that the sample was largely one of convenience. This is because although representative of global regions, the sample included countries for which data was available.

Additionally, being a global level study, we had to resort to variables that are coarse and upstream in nature and may not reveal as much as finely disaggregated variables would. For instance, the role of education being insignificant in this study would be counterintuitive to findings in the literature, because it is most found to be one of the primary determinants of health outcomes [24,25,26]. However, considering that literacy rate is a relatively coarse variable because of its broad definition, and considering that data is lacking to appropriately explore the effect of disaggregation of the literacy variable, there is a need to exercise caution in drawimg such a broad conclusion. Perhaps a disaggregate variable such as per cent population with different levels of education such as primary, secondary, and tertiary, would produce different results [26].

Furthermore, although the upstream variables presented in this study offers direction as to broad areas which may require the most focus, the results do not substitute detailed in-country studies that may be able to utilize more disaggregated and proximate variables. This is because such studies oftentimes are able to utilize fine-scale variables or data more in the context of an individual country’s economic, political, or environmental circumstances, which are likely to achieve results that will more precisely drive local interventions.

## Conclusions

Africa has generally been able to reduce its average U5MR during the MDGs era with help of improved socioeconomic conditions achieved in many countries, but many countries failed to meet the MDG target. There still remains a huge gap between Africa and rest of the world in terms of U5MR. Some studies have sought to illuminate important factors that should be considered on the global level to achieve the new target of 25 deaths in every 1000 births in the SDGs. However, in-country and regional peculiarities can derail one-size-fits-all approaches. This study addressed this for Africa to determine the main factors that account for the gap between the continent and the rest of the world.

First, it has revealed that with all things held constant, channeling resources towards providing access to basic healthcare may contribute most to bridging the U5MR gap between Africa and the rest of the world. Second, with all things considered, Africa will significantly benefit from interventions geared towards both the treatment and prevention of acute infectious diseases in the form of providing affordable maternal healthcare services and providing access to improved drinking water and sanitation. Additionally, increased educational programs aimed at mothers, especially adolescents, will help Africa bridge the U5MR gap towards achieving SDG number 3.

## Additional File

The additional file for this article can be found as follows:

10.29024/aogh.9.s1 Appendix 1. Variables Considered and their Respective Sources.
