# Supplementary material for: Bridging the Under-Five Mortality Gap for Africa in the Era of Sustainable Development Goals: An Ordinary Least Squares (OLS) Analysis
Source: Ann Glob Health. 2018 Apr 30;84(1):110–20. doi: 10.29024/aogh.9 (PMC6748240; doi:10.29024/aogh.9)
Supplement: Appendix 1. — Variables Considered and their Respective Sources. [file agh-84-1-9-s1.pdf]

Appendix 1

Variable	Hypothesis for Selection	Source
**U5MR**	This is the dependent variable of the study	World Development Indicators (WDI) database (http://data.worldbank.org/indicator)
**Gross National Income per Capita**	Many in-country studies have shown that income and socioeconomic status are imperative in determining child survivability.[27] GNIPC, based on purchasing power parity (PPP), obtained from WDI, was adopted as a proxy independent variable as an equivalent of these variables on the national level.	World Development Indicators (WDI) database (http://data.worldbank.org/indicator)
**Total Fertility Rate**	Many in-country studies of childhood mortality have revealed that there exist a symbiotic relationship between high fertility and childhood mortality rates.[28] For this reason, it is critical to consider fertility as an independent variable in this study. The WDI defines it as the total number of children a woman is likely to bear, assuming she lives to the end of her child bearing years, with the children born according to current age-specific fertility rates.	World Development Indicators (WDI) database (http://data.worldbank.org/indicator)
**Adolescent Fertility Rate**	Adolescent fertility rate is defined as the number of births per 1,000 women of age range 15–19 as according to WDI. Despite the risk of a high co-variation between total and adolescent fertility rate, adolescent fertility rate was included as a separate variable in the analysis. This is due to the fact that there exists the possibility of interaction between education and fertility, which may be more revealed only by adolescent fertility rate than total fertility rate, in determining childhood mortality rate. Many analysts have identified that the level of maternal education is critical in childhood mortality. Therefore, we assume that, all things being equal, an adolescent would have relatively fewer years of vital education compared to an adult. In this regard, our assumption is that, given any two countries with equal total fertility rate but differing adolescent fertility rate, *ceteris paribus*, the country with higher adolescent fertility will be worse off in terms of childhood mortality.[29]	World Development Indicators (WDI) database (http://data.worldbank.org/indicator)

**Total Female Employment to Population Ratio**	This variable measures the proportion of a country's female population of working age that is employed. Fifteen years old and above are generally considered as the working ages. Most studies have indicated that an employed female increases the income of her household, thereby increasing affordability of quality healthcare for her children.[23,30] For an inter-country level study, we considered total female employment to population ratio as an appropriate proxy variable with similar impact.	World Development Indicators (WDI) database (http://data.worldbank.org/indicator
**Per Cent Rural Population**	Literature from many sources have indicated that place of residence of a mother at the time of delivery, whether rural or urban, may be a determining factor of access (or lack thereof) to facilities and resources that could determine a child's chances of survival till the age of five.[31,32,33]	World Development Indicators (WDI) database (http://data.worldbank.org/indicator)
**Per Cent Population with Access to Improved Sanitation Facilities**	According to WDI, this variable is defined as the percentage of population that are likely to ensure that human excreta and human contact are separated in a hygienic manner. It is an important variable in the childhood mortality discourse as it is common knowledge, buttressed by research, that children are at a higher risk of mortality if they live in a household without access to improved sanitatary facilities.[34,35]	World Development Indicators (WDI) database (http://data.worldbank.org/indicator)
**Per Cent Population with Access to Improved Drinking Water**	The WDI defines this as percentage of national population with access to improved drinking water source such as piped water on premises and other drinking water sources such as public taps, boreholes, protected wells, rain water collection. Contact with waterborne pathogenic microorganisms is known to be a leading cause of diarrhea which claims the lives of many children under five years old.[5,36] For this reason, we viewed access to improved drinking water as a critical variable in our analysis.	World Development Indicators (WDI) database (http://data.worldbank.org/indicator)
**Per Capita Total Expenditure on Health**	Children have greater chance of survival if there is better access to quality healthcare provision.[37] To ascertain the effect of accessibility to healthcare for an inter-country level study, we selected total health expenditure per capita at average exchange rate in dollars as a proxy variable.	WHO Global Health Expenditure Database (http://apps.who.int/nha/database/Select/Indicators/en)
**Out-of-pocket Expenditure as a Per Cent of Total Health**	It is one of two variables selected to measure the effect of disaggregation of total health expenditure per capita. Many studies have revealed that individuals are less likely to access medical facilities and healthcare that may be vital to a child's survival if they have to pay from out-of-pocket.[38,39]	WHO Global Health Expenditure Database (http://apps.who.int/nha/database/Select/Indicators/en)

**Expenditure**		
**Government Expenditure on Health as a Per Cent of Total Health Expenditure**	This variable is the second selected to estimate the effect of disaggregation of the "total health expenditure per capita" variable. As an alternate to the previous case, it is the assumption of the study that the higher the government share is in the total expenditure on healthcare per person, the greater the likelihood that individuals may be willing to access healthcare, which will result in improved chances of survival of children.	WHO Global Health Expenditure Database (http://apps.who.int/nha/database/Select/Indicators/en).
**Per Cent Population Living under National Poverty Line**	This variable was viewed as very crucial to the analysis since most studies have shown that increased poverty is strongly associated with high child mortality.[37,40,41] This variable was chosen among many other poverty-related variables such as percent population living on less than $1.25 a day (PPP) and World Bank Multi-Dimensional Index for poverty, among others. We selected this variable because it is more individualized for each country, taking into consideration their unique circumstances and attributes, such as cost and standard of living.	World Development Indicators (WDI) (http://data.worldbank.org/indicator); CIA World Factbook (https://www.cia.gov/library/publications/resources/the-world-factbook/)
**Female Adult Literacy Rate**	According to the WDI, this variable is the percentage of female population above the age 15 who can read and write a short, simple statement on their daily life with understanding. Many researchers have investigated the influence of maternal education on in-country childhood mortality. We adopted female adult literacy rate as an equivalent of maternal education for inter-country under-five mortality rate analysis.	UNESCO (http://data.uis.unesco.org/Index.aspx?queryid=166); WDI (http://data.worldbank.org/indicator); CIA World Factbook (https://www.cia.gov/library/publications/resources/the-world-factbook/).
**Total Adult Literacy Rate**	As far as parental education level is concerned in the U5MR discourse, it is still contentious whether maternal education is more influential than father's literacy.[42,43] In spite of the potential of a high correlation between total adult literacy rate and female adult literacy rate, we still considered it as pertinent to keep total adult literacy rate as part of the analysis to compensate for the effect of male literacy rate.	UNESCO (http://data.uis.unesco.org/Index.aspx?queryid=166); WDI (http://data.worldbank.org/indicator); CIA World Factbook (https://www.cia.gov/library/publications/resources/the-world-factbook/).

References

27 **Schoeps A, Souares A, Niamba L, Diboulo E,** et al. Childhood mortality and its association with -household wealth in rural and semi-urban Burkina Faso. *Transactions of the Royal Society of -Tropical Medicine and Hygiene*. 2014; tru124: x–y. DOI: https://doi.org/10.1093/trstmh/tru124

28 **Rutstein SO** and **Winter R.** The effects of fertility behavior on child survival and child nutritional status: Evidence from the Demographic and Health Surveys 2006 to 2012; 2014.

29 **Singh L, Rai RK** and **Singh PK.** Assessing the -utilization of maternal and child health care among married adolescent women: Evidence from India. *Journal of Biosocial Science*. 2012; 44(01): 1–26. DOI: https://doi.org/10.1017/S0021932011000472

30 **Aseweh Abor P, Abekah-Nkrumah G, Sakyi K,** et al. The socio-economic determinants of maternal health care utilization in Ghana. *International -Journal of Social Economics*. 2011; 38(7): 628–648. DOI: https://doi.org/10.1108/03068291111139258

31 **Ettarh RR** and **Kimani J.** Determinants of under-five mortality in rural and urban Kenya. *Rural Remote Health*. 2012; 12(1812).

32 **Wang L.** Determinants of child mortality in LDCs: Empirical findings from demographic and health surveys. *Health Policy*. 2003; 65(3): 277–299. DOI: https://doi.org/10.1016/S0168-8510(03)00039-3

33 **World Health Organization (WHO).** Child -mortality indicators: Latest situation and change over time; 2015. http://www.who.int/gho/health_equity/outcomes/under5_mortality_text/en.

34 **Fink G, Günther I** and **Hill K.** The effect of water and sanitation on child health: Evidence from the demographic and health surveys 1986–2007. *-International Journal of Epidemiology*. 2011; 40(5): 1196–1204. DOI: https://doi.org/10.1093/ije/dyr102

35 **Günther I** and **Fink G.** Water and sanitation to reduce child mortality: The impact and cost of water and sanitation infrastructure. *World Bank Policy Research Working Paper Series*; 2011.

36 **Cheng JJ, Schuster-Wallace CJ, Watt S, -Newbold BK** and **Mente A.** An ecological quantification of the relationships between water, sanitation, and infant, child, and maternal mortality. *Environmental Health*. 2012; 11(1): 1–8. DOI: https://doi.org/10.1186/1476-069X-11-4

37 **Filmer D** and **Pritchett L.** Child mortality and public spending on health: How much does money -matter? *World Bank Publications*. 1997; 1864.

38 **Deloitte Center for Health Solutions.** Dig deep: Impacts and implications of rising out-of-pocket health care costs; 2014. https://www2.deloitte.com/content/dam/Deloitte/us/Documents/life-sciences-health-care/us-lchs-dig-deep-hidden-costs-112414.pdf.

39 **The Commonwealth Fund.** Too high a price: -Out--of-pocket health care costs in the United States; 2014. http://www.commonwealthfund.org/~/media/files/publications/issue-brief/2014/nov/1784_collins_too_high_a_price_out_of_pocket_tb_v2.pdf.

40 **Houweling TA, Kunst AE, Looman CW** and **-Mackenbach JP.** Determinants of under-5 -mortality among the poor and the rich: A -cross-national -analysis of 43 developing countries. Int. J. -*Epidemiology*. 2005; 34(6): 1257–1265. DOI: https://doi.org/10.1093/ije/dyi190

41 **Moser AK, Leon DA** and **Gwatkin DR.** How does progress towards the child mortality -millennium development goal affect inequalities between the poorest and least poor? Analysis of demographic and health survey data. *BMJ*. 2005; 331(1180): x–y.

42 **Breierova L** and **Duflo E.** The impact of education on fertility and child mortality: Do fathers really matter less than mothers? (No. w10513). *National Bureau of Economic Research*; 2004. DOI: https://doi.org/10.3386/w10513

43 **Macassa G, Ghilagaber G, Bernhardt E,** et al. Inequalities in child mortality in Mozambique: -Differentials by parental socio-economic position. *Social Science & Medicine*. 2003; 57(12): 2255–2264. DOI: https://doi.org/10.1016/S0277-9536(02)00545-2
